# Awareness of HPV, HPV vaccine and associated factors among male junior high school students in Zhejiang Province, China

**DOI:** 10.3389/fpubh.2026.1727798

**Published:** 2026-02-09

**Authors:** Xiang Zhao, Xuehai Zhang, Yue Xu, Yu Huang, Lei Wang, Qiaohong Lv, Suxian Wu

**Affiliations:** Department of Health Education, Zhejiang Provincial Center for Disease Prevention and Control, Hangzhou, China

**Keywords:** awareness, China, health education, HPV, HPV vaccine, junior high school, male students

## Abstract

**Objective:**

With HPV vaccines for males recently approved in China (2025), this study assessed awareness of HPV and HPV vaccine among junior high school boys in Eastern China, a population underrepresented in research.

**Methods:**

A cross-sectional study was conducted in Zhejiang Province in 2023 using multistage cluster sampling. Three cities were purposively selected; within each city, one urban district and one rural county were randomly chosen. A total of six junior high schools (one from each selected district/county) participated in the study, with students randomly sampled from each grade level. Participants completed an anonymous online questionnaire. Univariate and multivariate logistic regression analyses were performed to identify factors associated with awareness of HPV and the HPV vaccine among male junior high school students.

**Results:**

Among 1,786 male participants, awareness rates for HPV and the HPV vaccine were 24.7 and 30.7%, respectively. Multivariate analysis revealed factors significantly associated with HPV awareness: urban residence (aOR = 2.20, 95% CI: 1.57–3.08), knowing someone affected by cancer (aOR = 1.40, 95% CI: 1.02–1.92), cervical cancer awareness (aOR = 5.04, 95% CI: 3.48–7.28), HPV vaccine awareness (aOR = 21.31, 95% CI: 15.35–29.60), and concern for partner’s cervical cancer risk (aOR = 1.75, 95% CI: 1.23–2.49). Factors significantly associated with HPV vaccine awareness included: knowing someone affected by cancer (aOR = 1.58, 95% CI: 1.18–2.13), cervical cancer awareness (aOR = 2.43, 95% CI: 1.78–3.33), HPV awareness (aOR = 19.84, 95% CI: 14.39–27.36), concern for partner’s cervical cancer risk (aOR = 1.47, 95% CI: 1.47–2.91), perceiving being less likely to be infected with HPV (aOR = 1.48, 95% CI: 1.06–2.06), and having received school-based health education on HPV/vaccine (aOR = 2.27, 95% CI: 1.67–3.08).

**Conclusion:**

Awareness of HPV and HPV vaccine among junior high school boys in Zhejiang province is low. Targeted interventions, particularly school health programs emphasizing HPV and vaccine knowledge, male HPV risks, the benefits of vaccination, and partner protection, are crucial to improve vaccine uptake in this population.

## Backgrounds

Human papillomavirus (HPV), with over 200 known genotypes, is the most common sexually transmitted infection affecting both men and women (1). It is classified into low-risk and high-risk types based on oncogenic potential: low-risk HPV causes benign lesions such as genital and anal warts, whereas high-risk HPV is linked to cervical, vaginal, anal, penile, and oropharyngeal cancers. HPV causes around 5% of all human cancers (2). Although most HPV infections in men are asymptomatic and transient, persistent infection can lead to anogenital and oropharyngeal cancers, genital warts, and may also contribute to male infertility (3–6). Additionally, HPV-related head and neck cancers, especially oropharyngeal squamous cell carcinomas, have sharply increased among men in recent decades (7, 8). Notably, 40.2% of HPV-associated cancers from 2001 to 2017 in the United States occurred in men (6).

It is estimated that 91.3% of men will acquire HPV at some point in their lifetime (9). The prevalence of any genital HPV infection was 45.2% among men aged 18–69 years in the United States (10). In China, HPV prevalence varies by population: 10.5% among general males (11), 22.5% among men attending reproductive health clinics in Shanghai (12), and 52.5% among those visiting dermatology and venereology clinics in Beijing (13). Male HPV infection is strongly linked to infection in female partners—contributing to both initial and recurrent HPV infections in women (3) and potentially increasing their risk of cervical cancer (14, 15). Concordance of HPV subtypes between sexual partners is high: 83.5% of men whose female partners test positive for cervical HPV also test positive for genital HPV (16).

HPV vaccination is highly effective in preventing HPV-associated cancers, particularly when administered before sexual debut (6). Globally, it can prevent approximately 90% of cervical cancers and substantial proportions of other anogenital and oropharyngeal cancers (17). HPV vaccine trials in HPV-naïve men have demonstrated efficacy against HPV infection and related anogenital diseases, including genital warts and anal intraepithelial neoplasia, with efficacy rates as high as 89.9%. Long-term studies further show sustained protection, with over 90% efficacy against HPV-related external genital lesions for up to 10 years (18). Vaccinating males not only reduces their own disease burden but also enhances herd immunity, further lowering HPV infection and cervical cancer rates in females (19). The World Health Organization recommends including boys in HPV immunization programs where feasible (20), and 11 countries—including the United States, the United Kingdom, Australia, and South Korea—have already done so (21). Many Western nations implement school-based programs for 12–13-year-olds (22). In the U.S., quadrivalent and nonavalent HPV vaccines are recommended for males aged 9–26 years, with a national target of 80% adolescent vaccination coverage (23, 24).

In January and April 2025, Merck’s quadrivalent and nonavalent HPV vaccines received approval from the China National Medical Products Administration (NMPA) for multiple new indications, applicable to males aged 9–26 and 16–26, respectively, becoming the first and only quadrivalent and nonavalent HPV vaccines approved in China for use in males of the appropriate age group (25). Awareness of HPV and the HPV vaccine is associated with willingness to vaccinate and serves as a determinant of vaccination among students (26–28). Perceived risk of HPV infection related disease among male students can help to increase their willingness to be vaccinated (29). However, in China, male students have limited access to information about HPV and HPV vaccines, and are largely unaware of the physical harm caused by HPV infection and the benefits of vaccination (30, 31). A meta-analysis reported an overall HPV vaccine acceptance rate of 47.04% (95% CI: 39.23–54.93%) among general male populations (32). However, acceptance is markedly lower among Chinese male university students, with self-reported vaccination intentions ranging from only 23 to 42% (33, 34). This gap is largely attributed to low awareness of HPV-related risks in men and the widespread misconception that HPV is primarily a women’s health issue.

Understanding of knowledge levels and related factors regarding HPV and its vaccines among male adolescents in mainland China remains limited. First, research on HPV awareness in China has overwhelmingly focused on female populations, particularly university students (27, 35) and parents of primary and junior high school children (36), leaving male adolescents significantly underrepresented in the literature. Second, the limited studies that have examined males have primarily targeted general adult men (32) or university-aged cohorts (28, 33, 34), whose health literacy, sexual experience, and decision-making contexts differ substantially from those of early adolescents in junior high school. Third, evidence specific to male junior high school students remains scarce: to date, no study in mainland China has employed a multi-city, stratified sampling design to assess HPV and HPV vaccine awareness among this age group in an economically advanced province such as Zhejiang. This gap is especially critical now that China has recently approved HPV vaccines for males, creating an urgent need for robust, representative evidence to inform the development of effective, gender-inclusive school-based health education programs.

Therefore, it is necessary to conduct research into the level of knowledge and related factors regarding HPV and the HPV vaccine among male junior high school students.

## Methods

### Research subjects and methods

The research data comes from a survey of junior high school students in Zhejiang Province regarding their knowledge of HPV and HPV vaccines, and their willingness to be vaccinated. The inclusion criteria for study participants were junior high school students currently enrolled in Year 7–9 who voluntarily participated in the survey. The student will be excluded from the study if either the student or the parent declines to participate, or if the student was unable to participate due to health-related reasons at the time of the survey.

The sample size calculation formula for this study is $N=\frac{Z_{\alpha }^{2}\times p\times q}{d^{2}}$ . The *p* represents the proportion of respondents who have heard of HPV and the HPV vaccine, *q* = 1-*p*, the significance level is set at *α* = 0.05, *z*^2^_α_ = 1.96, and the permissible error is *d* = 0.1*p*. Based on prior studies, approximately 15% of junior high school students have heard of HPV and 18% have heard of the HPV vaccine (26, 37). Thus, *p* was conservatively estimated at 15% to ensure sufficient statistical power.

A survey framework and preliminary questionnaire were developed based on published literature from both domestic and international sources. Following consultations with six experts on HPV vaccination, we conducted individual, in-depth qualitative interviews with six male and six female junior high school students to gather their feedback on the questionnaire content and survey methodology. Based on this input, the questionnaire was iteratively revised and refined to produce the final version, accompanied by a survey manual. The content of the questionnaire included questions on demographic characteristics, knowledge of HPV and HPV vaccines, perception of HPV infection risk, acceptance of school-based HPV prevention education, and expectations.

A multistage stratified sampling design was employed, with selections at each stage performed using a random number table. From March to May 2023, the 11 cities in Zhejiang Province were divided into three categories—high, medium and general—based on their per capita GDP levels. One city was randomly selected from each category. Within the selected cities, one urban district and one county were randomly chosen, and one junior high school was randomly selected from each urban district and county. From each junior high school, 130 students will be randomly selected from each year group (Year 7, Year 8, and Year 9) within each class level. If insufficient students were available, an additional class was randomly selected.

An anonymous, self-administered online questionnaire survey was conducted by class teachers, who were provided with training. They then provided parents with a QR code for the questionnaire. After school, students scan the QR code provided by their parents to access the questionnaire. Each IP address can only complete the questionnaire once.

### Ethical considerations

Class teachers explained the purpose and significance of the survey, its methods, the privacy protection policy and how the information would be used to parents and students, respectively. They emphasised that participation in the survey was voluntary and that not participating would not have any negative consequences for students. Informed consent was obtained from parents and students. Participation in this survey is subject to the agreement of both the students and their parents. The Ethics Review Committee of the Zhejiang Provincial Centre for Disease Control and Prevention approved this study.

### Measurement

In this research, awareness of HPV and awareness of the HPV vaccine were defined by the survey questions ‘Have you ever heard of HPV?’ and ‘Have you ever heard of the HPV vaccine?’, respectively.

In the original questionnaire, responses to all knowledge assessment items were categorized as ‘correct’, ‘incorrect’, or ‘do not know’. For the purpose of analysis, ‘incorrect’ and ‘do not know’ responses were combined into a single category.

The measurement of the consequences of HPV infection consists of seven questions relating to HPV infection, which can lead to cervical, vaginal, penile, oral, anal, vulvar cancers and genital warts. Each question has three response options: ‘correct’, ‘incorrect’, or ‘do not know’. A correct response is assigned 1 point, while an incorrect or do not know response is assigned 0 points. The possible score range is 0–7 points. Based on the distribution trend, the measurement is divided into three groups: 0 points, 1–4 points, and 5–7 points. The Cronbach’s alpha value for this measurement is 0.939.

The measurement of the preventive efficacy of the HPV vaccine consists of three questions: 1. Can the HPV vaccine prevent HPV infection? 2. Can the HPV vaccine prevent various cancers caused by HPV? 3. Is HPV-induced cervical cancer a vaccination-preventable disease? The response options for all three questions are ‘correct’, ‘incorrect’, or ‘do not know’. One point is awarded for a correct response and zero for an incorrect response or ‘do not know’. The possible score range is 0–3. Based on the distribution trend, the scores are divided into four groups: 0 point, 1 point, 2 points, and 3 points. The Cronbach’s alpha value for this measurement is 0.715.

### Statistical analysis

After exporting the data from the online questionnaire survey, a statistical analysis was conducted using SPSS 26.0 software. Count data were described using case numbers, proportions or rates (%). Intergroup differences were analysed using the chi-square test. Factors associated with the awareness of HPV and HPV vaccine among junior high school males were analysed using a logistic regression model. Variables with a *p* value of less than 0.05 in the univariate analysis were included in the multivariate logistic regression model. We used backward elimination, setting *p* < 0.10 as the exclusion criterion, to achieve a parsimonious model while minimizing overfitting and multicollinearity. All demographic variables were forcibly included in the mode regardless of *p* value to ensure control for potential confounding. The relationship between the dependent and outcome variable was represented by the odds ratio (OR) and 95% confidence interval (CI). A *p-*value of less than 0.05 indicated a statistically significant difference.

## Results

### Demographic characteristics of male junior high school students

A total of 4,089 students were surveyed, of whom 1,786 were male. Among the male students, those in Years 7, 8, and 9 accounted for 36.1, 32.5, and 31.4%, respectively (Table 1). 97.1% of the students were of Han Chinese ethnicity, 38.1% were the only child, 68.6% came from rural areas, and 48.2% of fathers and 50.9% of mothers had an educational attainment level of junior high school or below 84.9% of male students came from families with average economic circumstances.

**Table 1:** Univariate analysis of demographic characteristics and awareness of HPV/HPV vaccine among junior high school male students.

Variable	Total	Awareness of HPV		Awareness of HPV vaccine	
	*N* = 1786	*N* = 442	*p-*value	*N* = 549	*p-*value
Year					
Year 7	644 (36.1%)	151 (23.4%)	0.449	195 (30.3%)	0.945
Year 8	581 (32.5%)	142 (24.4%)		181 (31.2%)	
Year 9	561 (31.4%)	149 (26.6%)		173 (30.8%)	
Ethnicity					
Han	1735 (97.1%)	427 (24.6%)	0.434	535 (30.8%)	0.606
Ethnic minority	51 (2.9%)	15 (29.4%)		14 (27.5%)	
Only child					
Yes	680 (38.1%)	182 (26.8%)	0.121	223 (32.8%)	0.140
No	680 (38.1%)	260 (23.5%)		326 (29.5%)	
Hometown type					
Rural	1,225 (68.6%)	284 (23.2%)	0.024	383 (31.3%)	0.476
Urban/town	561 (31.4%)	158 (28.2%)		166 (29.6%)	
Father’s education					
Junior high or below	860 (48.2%)	194 (22.6%)	<0.001	260 (30.2%)	<0.001
High school or above	828 (46.4%)	239 (28.9%)		277 (33.5%)	
Do not know	98 (5.5%)	9 (9.2%)		12 (12.2%)	
Mother’s education					
Junior high or below	909 (50.9%)	199 (21.9%)	<0.001	266 (29.3%)	<0.001
High school or above	772 (43.2%)	230 (29.8%)		268 (34.7%)	
Do not know	105 (5.9%)	13 (12.4%)		15 (14.3%)	
Family economic status					
Rich	81 (4.5%)	26 (32.1%)	0.077	29 (35.8%)	0.090
Average	1,516 (84.9%)	379 (25.0%)		474 (31.3%)	
Poor	189 (10.6%)	37 (19.6%)		46 (24.3%)	

### Univariate and multivariate regression analysis of junior high school boys’ awareness of HPV

A total of 442 junior high school boys were aware of HPV, giving an awareness rate of 24.7% (442 out of 1786). The chi-squared test showed significant differences in awareness of HPV between boys from different hometown types, and between those whose fathers and mothers had different levels of education (Table 1). HPV awareness was also influenced by whether anyone in their surroundings had cancer, whether they had heard of cervical cancer, the perceived likelihood of HPV infection, whether they were concerned that their sexual partner might have cervical cancer, whether their school conducted health education related to HPV and HPV vaccine, whether they believed it was necessary for their school to conduct such education, and whether they had heard of the HPV vaccine (Table 2). These variables were distributed differently among junior high school boys in terms of HPV awareness (*p* < 0.001).

**Table 2:** Univariate analysis of HPV and vaccine awareness among junior high school male students.

Variable	Total	Awareness of HPV		Awareness of HPV vaccine	
	*N* = 1786	*N* = 442	*p-*value	*N* = 549	*p*-value
Know someone with cancer					
No	1,169 (65.5%)	201 (17.2%)	<0.001	262 (22.4%)	<0.001
Yes	617 (34.5%)	241 (39.1%)		287 (46.5%)	
Heard of cervical cancer					
No	886 (49.6%)	56 (6.3%)	<0.001	105 (11.9%)	<0.001
Yes	900 (50.4%)	386 (42.9%)		444 (49.3%)	
Perceived likelihood of HPV infection					
Impossible/do not know	1,373 (76.9%)	284 (20.7%)	<0.001	357 (26.0%)	<0.001
Possible	413 (23.1%)	158 (38.3%)		192 (46.5%)	
Concern about partner getting cervical cancer					
Not concerned/do not know	1,426 (79.8%)	270 (18.9%)	<0.001	343 (24.1%)	<0.001
Concerned	360 (20.2%)	172 (47.8%)		206 (57.2%)	
School provided HPV/HPV vaccine health education					
No	727 (40.7%)	103 (14.2%)	<0.001	125 (17.2%)	<0.001
Yes	1,059 (59.3%)	339 (32.0%)		424 (40.0%)	
Belief in need for school HPV/HPV vaccine health education					
Unnecessary/do not know	337 (18.9%)	37 (11.0%)	<0.001	50 (14.8%)	<0.001
Necessary	1,449 (81.1%)	405 (28.0%)		499 (34.4%)	
Heard of HPV					
No	1,344 (75.3%)			176 (13.1%)	<0.001
Yes	442 (24.7%)			373 (84.4%)	
HPV infection consequences measurement^*^					
0	69 (15.6%)			43 (62.3%)	<0.001
1–6	200 (45.2%)			184 (92.0%)	
7	173 (39.1%)			146 (84.4%)	
HPV primarily sexually transmitted^*^					
Incorrect/do not know	130 (29.4%)			94 (72.3%)	<0.001
Correct	312 (70.6%)			279 (89.4%)	
Males can get HPV^*^					
Incorrect/do not know	213 (48.2%)			165 (77.%5)	<0.001
Correct	229 (51.8%)			208 (90.8%)	
Heard of HPV vaccine					
No	1,237 (69.3%)	69 (5.6%)	<0.001		
Yes	549 (30.7%)	373 (67.9%)			
HPV vaccine effectiveness measurement^#^					
0–1	154 (28.1%)	83 (53.9%)	0.001		
2	144 (26.2%)	94 (65.3%)			
3	251 (45.7%)	196 (78.1%)			
Males need HPV vaccination^#^					
Incorrect/do not know	295 (53.7%)	186 (63.1%)	0.008		
Correct	254 (46.3%)	187 (73.6%)			
Willingness for HPV vaccination^#^					
Unwilling/do not know	117 (21.3%)	70 (59.8)%	<0.001		
Willing	432 (78.7)%	303 (70.1%)			

^*^Among boys who heard of HPV (*n* = 442), this variable was not included in multivariate analysis.

^#^Among boys who heard of HPV vaccine (*n* = 549), this variable was not included in multivariate analysis.

Among boys who had heard of the HPV vaccine, those who were more aware of the vaccine’s effectiveness, knew that men needed to be vaccinated against HPV, and those who were more willing to be vaccinated, had a significantly higher awareness of HPV than those who were unaware of these facts.

Variables that were statistically significant in the univariate analysis were included in the multivariate analysis model to adjust for potential confounding. The results of the multivariate analysis of junior high school boys’ awareness of HPV showed that urban students than rural students (OR = 2.20, 95% CI: 1.57–3.08), those with someone in their surroundings, those who had cancer than those without (OR = 1.40, 95% CI: 1.02–1.92), and those who had heard of cervical cancer than those who had not (OR = 5.04, 95% CI: 3.48–7.28), those who had heard of the HPV vaccine than those who had not (OR = 21.31, 95% CI: 15.35–29.60), and those who were concerned about their sexual partners having cervical cancer than those who were not concerned or do not know (OR = 1.75, 95% CI: 1.23–2.49), were more likely to be aware of HPV (Table 3).

**Table 3:** Multivariate analysis of HPV and HPV vaccine awareness among junior high school male students.

Variable	Awareness of HPV		Awareness of HPV vaccine	
	Adjusted OR (95%CI)^*^	*p-*value	Adjusted OR (95%CI)^*^	*p-*value
Hometown type				
Rural (Ref)	1	<0.001		
Urban/town	2.20 (1.57–3.08)			
Know someone with cancer				
No (Ref)	1	0.039	1	0.002
Yes	1.40 (1.02–1.92)		1.58 (1.18–2.13)	
Heard of cervical cancer				
No (Ref)	1	<0.001	1	<0.001
Yes	5.04 (3.48–7.28)		2.43 (1.78–3.33)	
Heard of HPV				
No (Ref)			1	<0.001
Yes			19.84 (14.39–27.36)	
Heard of HPV vaccine				
No (Ref)	1	<0.001		
Yes	21.31 (15.35–29.60)			
Perceived likelihood of HPV infection				
Impossible/don’t know (Ref)			1	0.020
Possible			1.48 (1.06–2.06)	
Concern about partner getting cervical cancer				
Not concerned/don’t know (Ref)	1	0.002	1	<0.001
Concerned	1.75 (1.23–2.49)		2.07 (1.47–2.91)	
School provided HPV/HPV vaccine health education				
No (Ref)	1	0.076	1	<0.001
Yes	1.36 (0.97–1.91)		2.27 (1.67–3.08)	

^*^OR, odd ratio; CI, confidence interval.

### Univariate and multivariate regression analysis of junior high school boys’ awareness of HPV vaccine

Among the males, 549 were aware of the HPV vaccine, giving an awareness rate of 30.7% (549 out of 1786). The chi-square test showed an association between awareness of the HPV vaccine, and the educational level of the father and mother (Table 1), whether anyone in their surroundings had cancer, whether they had heard of cervical cancer, the perceived likelihood of HPV infection, whether they were concerned that their sexual partner might have cervical cancer, whether their school conducted health education related to HPV and the HPV vaccine, whether they thought their school should conduct such education, and whether they had heard of HPV (Table 2). The distribution of these variables differed statistically depending on whether or not the survey subjects were aware of the HPV vaccine (*p* < 0.005).

Among boys who have heard of HPV, those who are aware of the consequences of HPV infection, know that HPV is mainly transmitted through sexual activity, and understand that men can also be infected with HPV, are more likely to be aware of the HPV vaccine.

Variables that were statistically significant in the univariate analysis were included in the multivariate analysis model to adjust for potential confounding. The multivariate analysis revealed that the following factors were statistically associated with male students’ awareness of the HPV vaccine: having someone in their surroundings who had cancer (OR = 1.58, 95% CI: 1.18–2.13), having heard of cervical cancer (OR = 2.43, 95% CI: 1.78–3.33) and having heard of HPV (OR = 19.84, 95% CI: 14.39–27.36), being more concerned than not concerned/do not know about their sexual partner having cervical cancer (OR = 1.47, 95% CI: 1.47–2.91), believing they were less likely to be infected with HPV or do not know (OR = 1.48, 95% CI: 1.06–2.06) and having received health education about HPV and HPV vaccine at school (OR = 2.27, 95% CI: 1.67–3.08) (Table 3).

## Discussion

The proportion of boys in Zhejiang province who had heard of HPV and the HPV vaccine was 24.7 and 30.7%, respectively. Although this is higher than the 11.3 and 15.9% reported in a previous national survey of adolescent males (37), and the 15.9 and 19.4% reported among junior high school boys in Jinan, China (26), the level of awareness remains low. This may be due to the fact that HPV vaccination in mainland China was only available to females at the time of this survey, with little education targeting adolescent males. This may also be due to sociocultural and structural barriers. In China, sex education remains a sensitive topic, with limited discussion of HPV, vaccination or male-related sexual health risks (38). Parent–child communication about sexual topics is also very limited (27), and school-based health education varies considerably across regions.

Awareness of HPV and awareness of HPV vaccine are strongly correlated, with an adjusted OR of approximately 20. Among male students who have heard of HPV, those with higher knowledge scores about HPV related diseases, awareness that HPV can be sexually transmitted, and understanding that males can be infected with HPV, show a higher proportion of having heard of the HPV vaccine. Among students aware of HPV vaccine, factors such as higher scores in perceived vaccine efficacy, recognition that males also need vaccination, and willingness to receive the vaccine, were associated with awareness of HPV. These results indicate that better understanding and more knowledge about HPV is associated with better awareness of the HPV vaccine, and vice versa. Factors such as having heard of HPV and HPV vaccine (26), possessing knowledge about the vaccine (39), and receiving male-specific HPV-related information (27, 40) significantly increased vaccine acceptance. The higher the knowledge level of HPV and HPV vaccine, the stronger the intent and willingness to get vaccinated (41). To promote HPV vaccination among male students, efforts should not only focus on raising awareness of HPV and vaccine itself, but also emphasize HPV and vaccine related knowledge. This includes educating students about male-specific HPV-related diseases and the prophylactic benefits of vaccinating adolescent males, thereby enhancing HPV vaccine uptake in this population.

Among male students who had heard of HPV, only 51.8% were aware that males can also be infected with HPV. Similarly, among those aware of HPV vaccine, only 46.3% knew that males need HPV vaccination. Given that less than one-third of male students had heard of HPV or HPV vaccine, it is estimated that the actual awareness rates of these two aspects of knowledge are very low. In this study, only 23.1% of males perceived their own susceptibility to HPV infection. Students aware of HPV vaccine were more likely to recognize their infection risk compared to those unaware of the vaccine. Therefore, targeted educational interventions disseminating accurate information about HPV transmission in men and the preventive role of HPV vaccine for males need to be strengthened among male students.

We observed that male students from urban areas were 2.20 times more likely to have heard of HPV than those from rural areas. There is a possibility that urban male students are superior in terms of school resources, family background, and access to diverse health information compared to their rural counterparts. A Chinese study found that urban junior school students were more willing to take HPV vaccine than rural students (26). Therefore, HPV prevention education requires greater attention toward students from rural regions.

Awareness of cancer cases among acquaintances was positively associated with awareness of both HPV and the HPV vaccine, with adjusted OR of 1.4 and 1.6, respectively. Exposure to cancer narratives may reinforce students’ awareness of the severity of HPV-related diseases and the importance of prevention. Given that mainland China recently approved HPV vaccination for males, this finding supports incorporating cancer narratives, particularly those involving HPV-related cancers such as penile and oral cancers, into prevention and vaccination promotion strategies for adolescent males.

Students who had heard of cervical cancer were five times more likely to be aware of HPV and 2.4 times more likely to be aware of the HPV vaccine. While HPV prevention campaigns in China predominantly focus on cervical cancer in women, this study reveals that most participants scored low in recognizing HPV-related cancers affecting males (e.g., anal, oropharyngeal, penile cancers). This knowledge gap may directly contribute to low HPV vaccination willingness among males. Future educational interventions must strengthen male adolescents’ understanding of their own disease risks from HPV infection, explicitly emphasizing that male vaccination reduces partner transmission and lowers risks of both male-specific cancers and cervical cancer in female partners.

This study found that 20% of male students expressed concern about their partners developing cervical cancer, contrasting with a rate of 8.5% reported in a prior Jinan-based survey (26). Such concern is positively associated with awareness of HPV and HPV vaccination. Male students who understand the link between HPV infection and cervical cancer, along with the primary prevention role of vaccination, develop health-related concern for their partners. They may also worry that their own HPV infection could increase their partner’s cervical cancer risk. Although these boys are generally not yet sexually active, such anticipatory concern may lay the foundation for future supportive health behaviors, such as encouraging partners to receive HPV vaccination or undergo cervical cancer screening.

School-based health education serves as a critical catalyst for enhancing HPV knowledge and vaccine awareness, ultimately improving vaccination coverage in target populations (41, 42). Integrating HPV-specific content into middle school health curricula (41) and routine sexual education programs (42) demonstrates significant efficacy in elevating students’ knowledge levels and vaccine acceptability. In China, advancing sex education holds the potential to substantially increase HPV vaccination intent across all genders (26). Our findings underscore an urgent need for expanded school-based interventions focusing on HPV infection mechanisms and disease prevention strategies. Governments should strategically leverage schools as pivotal platforms to implement multifaceted education initiatives such as embedding HPV modules within comprehensive health courses and gender-inclusive sexual education to mitigate sexually transmitted infections, including HPV-related diseases.

This study employed a randomized cluster sampling method across six junior high schools in three cities of Zhejiang Province, China. The well-represented sample provides valuable insights into male junior high school students’ awareness of HPV and its vaccine, as well as associated factors in this region. This study has several limitations. Firstly, as this is a cross-sectional study, the results must be interpreted with caution due to the limitations of such surveys. Temporal ordering between variables cannot be established, so all reported associations should be interpreted as correlational rather than causal. Secondly, the questionnaire used in this study was designed by the research team and its reliability and validity were not verified before the study began. Thirdly, while the measurement instruments had acceptable internal consistency, they were not formally validated for construct validity prior to data collection. This restricts our ability to accurately assess whether the items captured the intended underlying constructs, meaning that findings relating to knowledge scores should be interpreted with caution. Fourthly, self-reported data may be subject to reporting bias due to social expectations. Fifthly, although the survey was anonymous and voluntary, classroom teachers’ involvement in distributing QR codes and overseeing the process may have created social pressure for students to participate, or to respond honestly. Finally, given that the study was conducted exclusively in Zhejiang Province, a high-income coastal region, the findings are not generalizable to other Chinese regions with differing socioeconomic conditions, health literacy levels, and school health education systems.

In summary, the findings of this study may inform the development of targeted educational guidance and intervention strategies to enhance HPV awareness and promote vaccination uptake among adolescent males in Zhejiang Province. Our research revealed that junior high school students in Zhejiang Province have limited knowledge of HPV, HPV vaccines, and related topics. HPV vaccines is currently being approved for use among male students in China. It is therefore crucial to implement educational programmes targeting male junior high school students to improve their knowledge. These programmes can incorporate education on the prevention of cancer and/or cervical cancer, can include information on how HPV is transmitted, HPV-related diseases, the role of vaccines in disease prevention, raising awareness of the risks of HPV infection and increasing students’ concern for their female partners’ risk of cervical cancer, with the aim of expanding vaccine coverage among male junior high school students.

## Data Availability

All of the data and materials that support the findings of this study are available from the corresponding author upon reasonable request. Requests to access the datasets should be directed to xzhao@cdc.zj.cn.

## References

[1] Bosch FX Broker TR Forman D Moscicki AB Gillison ML Doorbar J . Comprehensive control of human papillomavirus infections and related diseases. Vaccine. (2013) 31 Suppl 7:H1–H31. doi: 10.1016/j.vaccine.2013.10.003, 24332295 PMC7605442

[2] de Martel C Ferlay J Franceschi S Vignat J Bray F Forman D . Global burden of cancers attributable to infections in 2008: a review and synthetic analysis. Lancet Oncol. (2012) 13:607–15. doi: 10.1016/S1470-2045(12)70137-7, 22575588

[3] Zou K Huang Y Li Z. Prevention and treatment of human papillomavirus in men benefits both men and women. Front Cell Infect Microbiol. (2022) 12:1077651. doi: 10.3389/fcimb.2022.1077651, 36506029 PMC9729793

[4] Garolla A Pizzol D Bertoldo A Menegazzo M Barzon L Foresta C. Sperm viral infection and male infertility: focus on HBV, HCV, HIV, HPV, HSV, HCMV, and AAV. J Reprod Immunol. (2013) 100:20–9. doi: 10.1016/j.jri.2013.03.004 23668923

[5] Teng Q Niu M Liu Y Reng S. Harm of HPV infection in men and protection of men with HPV vaccination. Chin J Immunol. (2022) 38:507–14.

[6] Liao CI Francoeur AA Kapp DS Caesar MAP Huh WK Chan JK. Trends in human papillomavirus-associated cancers, demographic characteristics, and vaccinations in the US, 2001-2017. JAMA Netw Open. (2022) 5:e222530. doi: 10.1001/jamanetworkopen.2022.2530, 35294540 PMC8928005

[7] Pytynia KB Dahlstrom KR Sturgis EM. Epidemiology of HPV-associated oropharyngeal cancer. Oral Oncol. (2014) 50:380–6. doi: 10.1016/j.oraloncology.2013.12.019, 24461628 PMC4444216

[8] Sabatini ME Chiocca S. Human papillomavirus as a driver of head and neck cancers. Br J Cancer. (2020) 122:306–14. doi: 10.1038/s41416-019-0602-7, 31708575 PMC7000688

[9] Chesson HW Dunne EF Hariri S Markowitz LE. The estimated lifetime probability of acquiring human papillomavirus in the United States. Sex Transm Dis. (2014) 41:660–4. doi: 10.1097/OLQ.0000000000000193, 25299412 PMC6745688

[10] McQuillan G Kruszon-Moran D Markowitz LE Unger ER Paulose-Ram R. Prevalence of HPV in adults aged 18-69: United States, 2011-2014. NCHS Data Brief. (2017) 280:1–8. 28463105

[11] Wei F Yin K Wu X Lan J Huang S Sheng W, et a1. Human papillomavirus prevalence and associated factors in women and men in South China: a population-based study. Emerg Microbes Infect (2016) 5 1–8. doi: 10.1038/emi.2016.118 PMC5148022 27876782

[12] Abudukelimu N Li J Zhang T Wang X Xu Z Zhu Q. Analysis of HPV infection and genotype distribution among 1658 male reproductive health outpatients. Fudan Univ J Med Sci. (2024) 51:1–7.

[13] Shao L Zhang T Yao B. Subtypes analysis of human papilloma virus infection in 1359 male patients and analysis of differences in male and female infection. Acad J Chin PLA Med Sch. (2022) 43:55–9. doi: 10.3969/j.issn.2095-5227.2022.01.012

[14] Wang G Gu H Lin F Hu Y. The analysis of HPV infection of genitals among heterosexual couples. Chin J Gen Practise. (2020) 18:1691–4.

[15] Hu J Ji L Li P Ni X Huang Y Tao J . Genital HPV prevalence, follow-up and persistence in males and HPV concordance between heterosexual couples in Wenzhou, China. Infect Drug Resist. (2022) 15:7053–66. doi: 10.2147/IDR.S387226, 36483141 PMC9725929

[16] Pan L Ma J Zhang F Pan F Zhao D Zhang X. HPV infection of the external genitalia in men whose female partners have cervical HPV infection. Zhonghua Nan Ke Xue Za Zhi. (2018) 24:516–9. doi: 10.13263/j.cnki.nja.2018.06.006 30173456

[17] de Sanjosé S Serrano B Tous S Alejo M Lloveras B Quirós B . Burden of human papillomavirus (HPV)-related cancers attributable to HPVs 6/11/16/18/31/33/ 45/52 and 58. JNCI Cancer Spectr. (2019) 2:pky045. doi: 10.1093/jncics/pky045 31360870 PMC6649711

[18] Rosado C Fernandes ÂR Rodrigues AG Lisboa C. Impact of human papillomavirus vaccination on male disease: a systematic review. Vaccines (Basel). (2023) 11:1083. doi: 10.3390/vaccines11061083, 37376472 PMC10302589

[19] Schmeler KM Sturgis EM. Expanding the benefits of HPV vaccination to boys and men. Lancet. (2016) 387:1798–9. doi: 10.1016/S0140-6736(16)30314-2, 27203488

[20] Nyitray AG Carvalho da Silva RJ Baggio ML Lu B Smith D Abrahamsen M . Age-specific prevalence of and risk factors for anal human papillomavirus (HPV) among men who have sex with women and men who have sex with men: the HPV in men (HIM) study. J Infect Dis. (2011) 203:49–57. doi: 10.1093/infdis/jiq021, 21148496 PMC3086435

[21] World Health Organization. Human papillomavirus vaccines: WHO position paper, May 2017. Wkly Epidemiol Rec. (2017) 92:241–68. doi: 10.1016/j.vaccine.2017.05.069 28530369

[22] Bjerke RD Laake I Feiring B Aamodt G Trogstad L. Time trends in HPV vaccination according to country background: a nationwide register-based study among girls in Norway. BMC Public Health. (2021) 21:854. doi: 10.1186/s12889-021-10877-8, 33941126 PMC8091748

[23] Yu Y Reng Y Niu H. Correlation of human papillomavirus (HPV) and HPV vaccines with male reproductive health. Zhonghua Nan Ke Xue Za Zhi. (2019) 25:49–753. 32227721

[24] The American Cancer Society. (2022) Mission: HPV Cancer free. Available online at: https://www.cancer.org/healthy/hpv-vaccine.html (Accessed April 16, 2025).

[25] Zhou Wenyun. (2025) The nine-valent HPV vaccine has been approved for administration to males aged 16–26 in China. Available online at: https://baijiahao.baidu.com/s?id=1829366987608337298&wfr=spider&for=pc (Accessed July 20, 2025).

[26] Xue L Hu W Zhang H Xie Z Zhang X Zhao F . Awareness of and willingness to be vaccinated by human papillomavirus vaccine among junior middle school students in Jinan, China. Hum Vaccin Immunother. (2018) 14:404–11. doi: 10.1080/21645515.2017.1393132, 29048994 PMC5806637

[27] Chen G Wu B Dai X Zhang M Liu Y Huang H . Gender differences in knowledge and attitude towards HPV and HPV vaccine among college students in Wenzhou, China. Vaccines (Basel). (2021) 10:10. doi: 10.3390/vaccines10010010, 35062671 PMC8779512

[28] Dai Z Si M Su X Wang W Zhang X Gu X . Willingness to human papillomavirus (HPV) vaccination and influencing factors among male and female university students in China. J Med Virol. (2022) 94:2776–86. doi: 10.1002/jmv.27478, 34825712 PMC9299831

[29] Xu Y Wang W Cheng C Yang L Xing C Yang X . Factors influencing HPV vaccination willingness among male college students in Jinan according to the health belief model. Sci Rep. (2025) 15:30369. doi: 10.1038/s41598-025-16299-5, 40830565 PMC12365129

[30] Wang S Han B Wan Y Liu J Zhao T Liu H . Do male university students know enough about human papillomavirus (HPV) to make informed decisions about vaccination? Med Sci Monit. (2020) 26:e924840. doi: 10.12659/MSM.924840, 32603317 PMC7346751

[31] Fu SJ. Cognitive investigation of human papillomavirus in 1362 junior high school male students (master's thes). Chongqing: Chongqing Medical University (2023).

[32] Shen F Du Y Cao K Chen C Yang M Yan R . Acceptance of the human papillomavirus vaccine among general men and men with a same-sex orientation and its influencing factors: a systematic review and meta-analysis. Vaccine. (2023) 12:16. doi: 10.3390/vaccines12010016, 38250829 PMC10819436

[33] Ran H Chen Y Gao J Guo H Peng S. Low awareness of HPV infection and willingness of HPV vaccination among Chinese male college students in the east of China. Front Public Health. (2022) 10:971707. doi: 10.3389/fpubh.2022.971707, 36203657 PMC9531242

[34] Zhang H Tang MY Tian XF. Investigation of HPV vaccine awareness and vaccination willingness among male students in private college based on model of TAM-TPB. Occup Health (Lond). (2022) 38:2260–5.

[35] Bai Y Ip P Chan K Ngan H Yip P. HPV vaccination intentions of female students in Chinese Universities: a systematic literature review and Meta-analysis. Int J Environ Res Public Health. (2022) 19:10207. doi: 10.3390/ijerph191610207, 36011838 PMC9407966

[36] Cui M Wang Y Liu Z Liu C Niu T Zhou D . The awareness and acceptance of HPV vaccines among parents of primary and junior high school students in China: a meta-analysis. Infect Med (Beijing). (2023) 2:273–82. doi: 10.1016/j.imj.2023.11.003, 38205181 PMC10774669

[37] Zhang X Wang Z Ren Z Li Z Ma W Gao X . HPV vaccine acceptability and willingness-related factors among Chinese adolescents: a nation-wide study. Hum Vaccin Immunother. (2021) 17:1025–32. doi: 10.1080/21645515.2020.1812314, 33121330 PMC8018488

[38] Zhang L Li X Shah IH Baldwin W Stanton B. Parent-adolescent sex communication in China. Eur J Contracept Reprod Health Care. (2007) 12:138–47. doi: 10.1080/13625180701300293, 17559012

[39] Zhao X Huang Y Lv Q Wang L Wu S Wu Q. Knowledge, awareness, and correlates of HPV vaccine acceptability among male junior high school students in Zhejiang Province, China. Hum Vaccin Immunother. (2024) 20:2357238. doi: 10.1080/21645515.2024.2357238, 38869047 PMC11178271

[40] Bonafide KE Vanable PA. Male human papillomavirus vaccine acceptance is enhanced by a brief intervention that emphasizes both male-specific vaccine benefits and altruistic motives. Sex Transm Dis. (2015) 42:76–80. doi: 10.1097/OLQ.0000000000000226 25585065

[41] Zhang X Liu CR Wang ZZ Ren ZF Feng XX Ma W . Effect of a school-based educational intervention on HPV and HPV vaccine knowledge and willingness to be vaccinated among Chinese adolescents: a multi-center intervention follow-up study. Vaccine. (2020) 38:3665–70. doi: 10.1016/j.vaccine.2020.03.032, 32245644

[42] Liu CR Liang H Zhang X Pu C Li Q Li QL . Effect of an educational intervention on HPV knowledge and attitudes towards HPV and its vaccines among junior middle school students in Chengdu, China. BMC Public Health. (2019) 19:488. doi: 10.1186/s12889-019-6823-0, 31046722 PMC6498581

